# Detection of BRCA1/2 pathogenic variants in patients with breast and/or ovarian cancer and their families. Analysis of 3,458 cases from Lower Silesia (Poland) according to the diagnostic algorithm of the National Cancer Control Programme

**DOI:** 10.3389/fgene.2022.941375

**Published:** 2022-09-12

**Authors:** Anna Doraczynska-Kowalik, Dagmara Michalowska, Rafal Matkowski, Ewelina Czykalko, Dorota Blomka, Mariola Semeniuk, Mariola Abrahamowska, Gabriela Janus-Szymanska, Paulina Mlynarczykowska, Bartlomiej Szynglarewicz, Ireneusz Pawlak, Adam Maciejczyk, Izabela Laczmanska

**Affiliations:** ^1^ Lower Silesian Oncology, Pulmonology and Hematology Center, Wroclaw, Poland; ^2^ Department of Genetics, Wroclaw Medical University, Wroclaw, Poland; ^3^ Department of Oncology, Wroclaw Medical University, Wroclaw, Poland

**Keywords:** HBOC, BRCA1/2, NGS, diagnostics, algorithm

## Abstract

Breast and ovarian cancers are among the most common malignancies in the female population, with approximately 5–10% of cases being hereditary. *BRCA1* and *BRCA2* with other homologous recombination genes are the most tested genes in hereditary breast and ovarian cancer (HBOC) patients. As next-generation sequencing (NGS) has become a standard and popular technique, such as for HBOC, it has greatly simplified and accelerated molecular diagnosis of cancer. The study group included 3,458 HBOC patients or their relatives from Lower Silesia (Poland) (a voivodeship located in south-west Poland inhabited by 2.9 million people). All patients were tested according to the recommendations from the National Cancer Control Programme of the Ministry of Health for the years 2018–21. We tested 3,400 patients for recurrent pathogenic variants for the Polish population: five *BRCA1* founder variants (c.5266dup, c.181T>G, c.4035del, c.3700_3704del, and c.68_69del), two *PALB2* variants (c.509_510del, c.172_175del) and three *CHEK2* variants [c.1100del, c.444+1G>A, g.27417113-27422508del (del5395)]. Next 260 patients from the study group were chosen for the *BRCA1/2* NGS panel, and additionally selected marker pathogenic variants were tested using Sanger sequencing and MLPA methods in 45 and 13 individuals, respectively. The analysis of *BRCA1/2* in the 3,458 patients with HBOC or their relatives revealed 144 carriers of 37 different pathogenic variants (22 in *BRCA1* and 15 in *BRCA2*). Among all detected variants, 71.53% constituted founder pathogenic *BRCA1* variants. Our study has revealed that for the Lower Silesian population, the first-line *BRCA1/2* molecular test may be limited to only three variants in *BRCA1*—c.5266dup, c.181T>G, and c.4035del—but the aim should be to provide a full screening test of HBOC critical genes. The key and still growing role of molecular diagnostics of neoplasms, which includes HBOC, is undeniable. Therefore, it is necessary to provide complete and optimal therapeutic and prophylactic algorithms in line with current medical knowledge.

## Introduction

Breast cancer is the most common malignancy in the worldwide female population, and with about two million new cases yearly, it represents one in four cancer diagnoses in women (Cardoso et al., 2019). The lifetime risk of developing breast cancer is estimated at approximately 12% for every woman (Momenimovahed and Salehiniya 2019). Moreover, the incidence rate for breast cancer is globally on the rise, especially in developed countries, probably due to the rising detection efficiency and population ageing (Ahmad 2019).

Ovarian cancer is the seventh most common malignancy in the worldwide female population, with about 295,500 new cases yearly. It represents 3.4% of cancer diagnoses in women. The lifetime risk for ovarian cancer development is estimated at about 2.7% in each woman. Ovarian cancer is thus far rarer than breast cancer, and it is even rarer than such gynaecological malignancies as cervical cancer and endometrial cancer. However, ovarian cancer is estimated to be three times more lethal than breast cancer, and it has the worst prognosis among all of the gynaecological cancers (Momenimovahed and Salehiniya 2019).

Because of their crucial role in the DNA double-strand break repair process *via* homologous recombination (HR), *BRCA1* and *BRCA2* (*BRCA1/2*) are among the most tested genes in patients with breast, ovarian, prostate, or pancreatic cancer (Yamamoto and Hirasawa 2021). Together with other HR genes—*ATM*, *BARD1*, *BRIP1*, *CHEK2*, *NBS1*, *PALB2*, *RAD51C*, and *RAD51D*—and also genes from other biological pathways such as PTEN, STK11, and TP53, BRCA1/2 are usually examined using next-generation sequencing (NGS) multigene panels in hereditary breast and ovarian cancer (HBOC) patients (McAlarnen et al., 2021; Yamamoto and Hirasawa 2021).

The cumulative risk of breast cancer development by the age of 80 years for *BRCA1* pathogenic variant carriers is estimated at 72% and of ovarian cancer at 44% and for *BRCA2* at 69% and 17%, respectively (Kuchenbaecker et al., 2017). The highest number of cancer cases is observed in patients between the ages of 30 and 40 years for breast cancer and 40 and 50 years for ovarian cancer. Also, higher risk is observed for patients with a large number of first- and second-degree relatives with breast cancer (Kuchenbaecker et al., 2017).

Approximately 5–10% of breast and ovarian cancers are hereditary. Because *BRCA1/2* pathogenic variants account for about 80% of all damaging alterations, it is logical to start the examination with these genes, especially in countries with founder pathogenic variants (founder mutations) present in the population (Kuchenbaecker et al., 2017; Rebbeck et al., 2018; McAlarnen et al., 2021). In Poland (as in Ashkenazi Jews in Israel, or in many countries in Europe) three main *BRCA1* founder pathogenic variants are present, namely, c.5266dup, c.181T>G, and c.4035del, and it is estimated that they may constitute 80–90% of all *BRCA1/2* pathogenic variants (Janavičius 2010). Hence, it is economically justified to examine patients with suspected HBOC beginning with the most common variants and, after the first screening study, to select patients for the more expensive and technically demanding NGS exon sequencing or multigene panel sequencing.

NGS, employing massive parallel sequencing, has become a standard molecular technique in cancer diagnostics. The widespread use of NGS is primarily due to high throughput, relatively short testing time, the ability to test multiple samples in one experiment, and the ever lower costs and user-friendly software for data analysis (Zhong et al., 2021). Whole-exome, whole-transcriptome, or whole-genome sequencing make it possible to identify genetic variations and find new markers and new biochemical pathways critical for this complex disease, while NGS gene panels designed for specific tumours provide data for cancer diagnosis and prognosis, as well as for treatment and prophylaxis strategies (Zhong et al., 2021). Therefore, NGS is now one of the most important elements in precision oncology. The widespread use of NGS in Poland is still limited by the relatively high costs of performing the test in relation to the government reimbursement and the lack of a sufficient quantity of specialized equipment and laboratory workers trained in this technique.

To our knowledge, this is the first such comprehensive report on the results obtained in the National Cancer Control Programme of the Ministry of Health for the years 2018–21 in Poland.

## Materials and methods

### Patients

The study group included 3,458 patients (82.94%) or their relatives (17.06%) from Lower Silesia (south-west province of Poland, with approximately 2.9 million population in 2019). The mean age of the patients was 54.7± 15.15 years (the youngest examined person was 18 years old, while the oldest was 96 years old). The median age was 55 years. There were 131 men in the study group (3.79%).

The patients were referred for a consultation with a clinical geneticist regarding hereditary breast and ovarian cancer predisposition due to such conditions as breast cancer diagnosis, ovarian cancer diagnosis, breast and/or ovarian cancer diagnosis in a close relative, HBOC-related mutation identified in the family, or *BRCA1/2* mutation detected in the NGS test performed on tumour DNA (tDNA) isolated from the ovarian cancer cells. The most common reason for referral was breast cancer (64% of patients), predominantly luminal infiltrating ductal carcinoma (NOS, ER positive, PR positive, and HER-2 negative) diagnosed at the Breast Unit of the Lower Silesian Oncology, Pulmonology and Hematology Centre, Wrocław, Poland.

**FIGURE 1:**
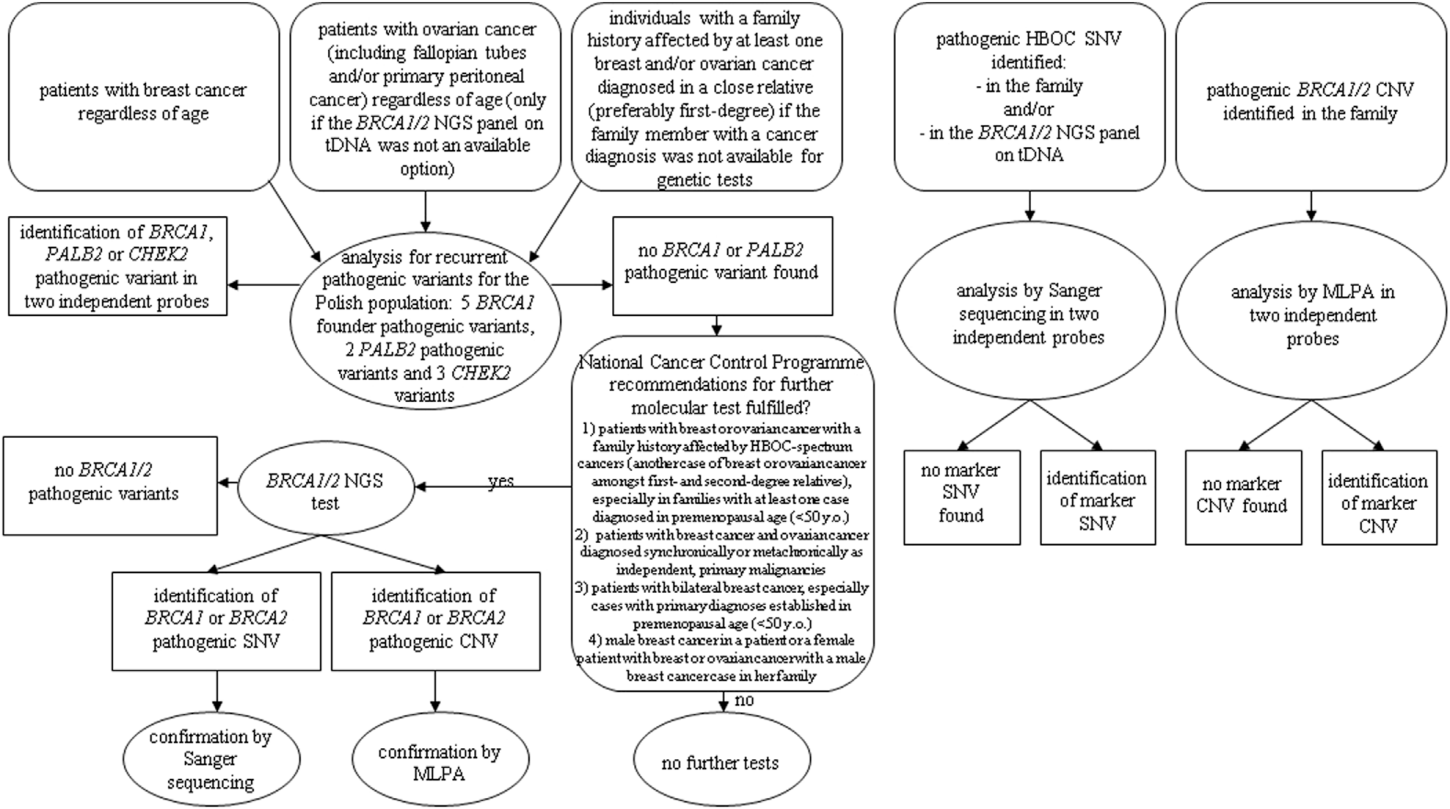
Diagnostic algorithm according to National Cancer Control Programme of the Ministry of Health for the years 2018–21.

According to the recommendations from the National Cancer Control Programme of the Ministry of Health for the years 2018–21, as the first-line HBOC genetic tests, we used the analysis for recurrent pathogenic variants for the Polish population: five *BRCA1* founder pathogenic variants (c.5266dup, c.181T>G, c.4035del, c.3700_3704del, and c.68_69del), two *PALB2* pathogenic variants (c.509_510del and c.172_175del), and three *CHEK2* variants [c.1100del, c.444+1G>A, and g.27417113-27422508del (del5395)] in 3,400 patients. For genetic tests analysing Polish recurrent HBOC-related mutations, we referred patients who met at least one of the following criteria: 1) patients with breast cancer regardless of age, 2) patients with ovarian cancer (such as fallopian tubes and/or primary peritoneal cancer) regardless of age (only if the *BRCA1/2* NGS panel on tDNA was not an available option), and 3) individuals with a family history affected by at least one breast and/or ovarian cancer diagnosed in a close relative (preferably first-degree), if the family member with a cancer diagnosis was not available for genetic tests (Figure 1).

In the cases with no *BRCA1* pathogenic variants found in the first-line genetic test, we reanalysed the pedigree and clinical data to select patients for the second-line *BRCA1/2* NGS test using the following criteria: 1) patients with breast or ovarian cancer with a family history affected by HBOC-spectrum cancers (another case of breast or ovarian cancer amongst first- and second-degree relatives), especially in families with at least one case diagnosed in premenopausal age (<50 years old), 2) patients with breast cancer and ovarian cancer diagnosed synchronically or metachronically as independent, primary malignancies, 3) patients with bilateral breast cancer, especially cases with primary diagnoses established in premenopausal age (<50 years old), and 4) male breast cancer in a patient or a female patient with breast or ovarian cancer with a male breast cancer case in her family.

According to the abovementioned criteria, after excluding founder mutations, we selected 260 (7.65%) patients for the *BRCA1/2* NGS test (Figure 1).

Additionally, we performed genetic tests aimed at selected marker pathogenic single-nucleotide variations (SNVs) and copy number variations (CNVs) in 45 and 13 individuals, respectively, with a specific HBOC-related pathogenic variant identified in the family and for patients with a particular pathogenic *BRCA1/2* variant found in the NGS test performed on tDNA isolated from ovarian cancer cells (nine patients) (Figure 1).

All patients signed an informed consent form before the genetic test. This study was performed in line with the principles of the Declaration of Helsinki. Approval was granted by the Ethics Committee of Wroclaw Medical University (No. 229/2022).

### DNA isolation

Genomic DNA (gDNA) was isolated from 300 µL of fresh whole blood using the Maxwell RSC Blood DNA Kit (Promega Corporation) according to the manufacturer’s protocol on a Maxwell RSC instrument (AS4500, Promega Corporation). After extraction, the DNA samples were quantified using the fluorometric method, using Quantus Fluorometer (Promega) and QuantiFluor ONE dsDNA System (Promega). The purity of DNA was determined on the NanoPhotometer N60 (Implen).

### Founder *BRCA1* pathogenic variants’ genotyping

Founder pathogenic variants *BRCA1*:c.5266dup (p.Gln1756fs), *BRCA1*:c.181T>G (p.Cys61Gly), and *BRCA1*:c.4035del (p.Glu1346fs) were detected by the PCR–restriction fragment length polymorphism (PCR-RFLP) method.

For identification of the *BRCA1* c.5266dup (p.Gln1756fs) variant, the Rohlfs et al. (1997) method with some modifications was used. An undigested 273-bp PCR product indicated the absence of a pathogenic variant, while digested 250-bp and 23-bp products indicated the presence of the *BRCA1*:c.5266dup variant (Rohlfs et al., 1997).

For the identification of *BRCA1* c.181T>G (p.Cys61Gly) and c.4035del (p.Glu1346fs) variants, RFLP together with the allele-specific amplification PCR (ASA-PCR) method with some modifications described by Górski et al. (2004) were used. An undigested 237-bp PCR product indicated the absence of a pathogenic variant *BRCA1*:c.181T>G, while digested 237-bp, 156-bp, and 80-bp products indicated the presence of examined variants. The pathogenic variant *BRCA1*:c.4035del was indicated by a 500-bp PCR product.

The pathogenic variants in the *BRCA1* gene, c.3700_3704del (p.Val1234fs) and c.68_69del (p.Glu23fs), were detected by the ASA-PCR method with originally designed primers (Supplementary Table). The PCR was conducted using Qiagen Multiplex PCR Master Mix (Qiagen) according to the manufacturer’s instructions. The temperature profile of PCR was as follows: initial denaturation at 95°C for 15 min; 25 cycles of denaturation at 94 °C for 30 s, annealing at 62°C for 90 s, and elongation at 72°C for 90 s. The final cycle was followed at 60°C for 30 min. Electrophoretic separation of PCR products was carried out using a 3500xL Genetic Analyzer (Applied Biosystems). The pathogenic variant *BRCA1*:c.3700_3704del was indicated by two products of 75 and 70-bp (a product with a length of 75-bp indicates the absence of the variant), whereas *BRCA1*:c.68_69del was indicated by 94-bp and 92-bp PCR products (a product with a length of 94-bp indicates the absence of the variant).

### Library preparation and next-generation sequencing

The detection of sequence variants in the *BRCA1* and *BRCA2* genes was performed using the Devyser BRCA kit (Devyser AB, Sweden) according to the manufacturer’s instructions. The Devyser BRCA kit is based on multiplex PCR amplification and allows detection of SNVs and indels and the quantitative detection of exon-spanning CNVs, all appearing in the coding regions and adjacent exon–intron boundaries in the *BRCA1* and *BRCA2* genes. The kit enables determination of all 22 coding exons of the *BRCA1* transcript NM_007294.4 and all 26 coding exons of the *BRCA2* transcript NM_000059.3. The sequencing reaction was carried out using the Illumina MiSeq System and MiSeq Reagent Micro Kit v2 (300 cycles) (Illumina).

### Bioinformatic/data analysis

Primary data analysis (cluster density, cluster passing filter, estimated yield, and Q30 score) was done directly in the MiSeq instrument. Secondary data analysis was performed using Amplicon Suite Software (SmartSeq) using generated MiSeq FASTQ files. The software enables alignment to the human reference genome hg19 (Genome Reference Consortium GRCh37). The analysis was based on the reference transcript sequences such as NM_007294.4 for *BRCA1* and NM_000059.3 for *BRCA2.* The optimal required coverage of amplicons was 200× in the case of analysis of SNVs, indels, and CNVs. The minimum required coverage was 60× in the case of analysis of only SNVs and indels. The samples with variant allele fraction (VAF) <20% were excluded from further analysis. CNV computation was based on the ratio of the number of reads with both intra-sample and inter-sample normalization within a run.

### Variant assessment

Variants were classified according to “Standards and Guidelines for the Interpretation of Sequence Variants: A Joint Consensus Recommendation of the American College of Medical Genetics and Genomics and the Association for Molecular Pathology” (Richards et al., 2015). All variants were classified as benign, likely benign, variants of uncertain significance (VUS), likely pathogenic, and pathogenic, class 1–5, respectively. Clinical classification was performed using publicly available databases such as ClinVar (National Center for Biotechnology Information, https://www.ncbi.nlm.nih.gov/clinvar/), BRCA Database ARUP Laboratories (https://arup.utah.edu/database/BRCA/Variants/BRCA1.php), Varsome (https://varsome.com/), and Franklin by Genoox (https://franklin.genoox.com/clinical-db/home). *In silico* functional predictions were performed using varSEAK (Splice Site Prediction, https://varseak.bio/index.php); Mutalyzer (https://v3.mutalyzer.nl/), PolyPhen-2 (Harvard University, http://genetics.bwh.harvard.edu/pph2/), and MutationTaster (http://www.mutationtaster.org). A probability of pathogenicity of single-nucleotide substitutions was assessed by the database PRIORS (http://priors.hci.utah.edu/PRIORS/). A literature search using PubMed (National Library of Medicine, https://pubmed.ncbi.nlm.nih.gov/), LitVar (https://www.ncbi.nlm.nih.gov/CBBresearch/Lu/Demo/LitVar/#!?query=), and Mastermind by Genomenon (Comprehensive Genomic Search Engine, https://mastermind.genomenon.com/) was applied to support variant assessment.

### Multiplex ligation-dependent probe amplification

The multiplex ligation-dependent probe amplification (MLPA) test was offered to patients with uninformative CNVs in NGS (six cases) or the presence of a pathogenic CNV in a close relative (seven cases).

CNVs in *BRCA1* and *BRCA2* were detected by MLPA using SALSA MLPA Probemix P002 *BRCA1* or SALSA MLPA Probemix P045 *BRCA2/CHEK2* (MRC-Holland, Amsterdam, The Netherlands) according to the manufacturer’s instructions. The PCR products were separated on 3500xL Genetic Analyzer (Applied Biosystems). The data analysis was performed using the Coffalyser.NET software (MRC-Holland, Amsterdam, The Netherlands).

### Sanger sequencing

Of the 45 patients qualified for Sanger sequencing, 32 were referred for the presence of a pathogenic variant in a close relative, 9 for the presence of a pathogenic variant in ovarian cancer tissue, and 4 for previously unconfirmed result from another medical unit.

All primers were designed using Primer-BLAST (https://www.ncbi.nlm.nih.gov/tools/primer-blast/index.cgi). The PCR was conducted using Hot Start Taq DNA Polymerase (Qiagen) according to the manufacturer’s instructions. Next, the PCR products were purified using Exonuclease I (20 units/µl) (Thermo Fisher Scientific) and FastAP Thermosensitive Alkaline Phosphatase (1 unit/µl) (Thermo Fisher Scientific). Sequencing was carried out using the BigDye Terminator v3.1 Cycle Sequencing Kit (Thermo Fisher Scientific, United States) according to the manufacturer’s instructions on 3500xL Genetic Analyzer (Applied Biosystems). The sequence data were analysed *via* FinchTV software (Geospiza Inc.).

Patients with the presence of pathogenic or likely pathogenic variants which were diagnosed using NGS and required confirmation using MLPA or Sanger sequencing were excluded from the MLPA and Sanger sequencing group in this study.

## Results

**FIGURE 2:**
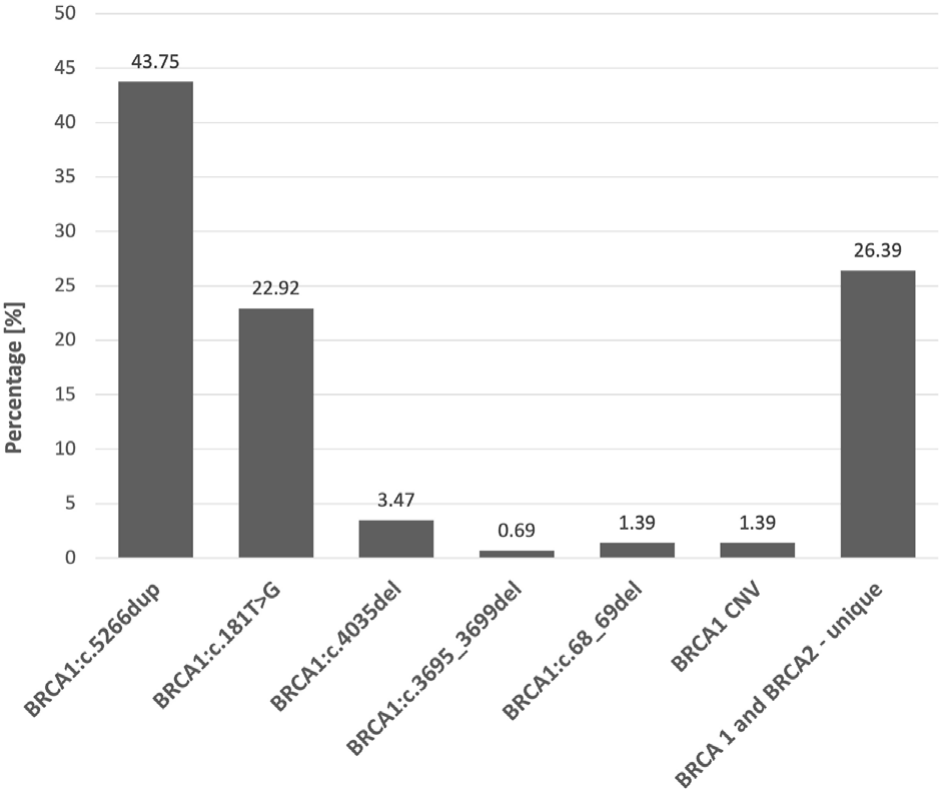
Percentage share of of all 144 detected Class 5 and 4 *BRCA1/2* variants.

The analysis of *BRCA1/2* genes in 3,458 patients with breast or ovarian cancer or their relatives revealed 144 carriers of pathogenic variants. Thirty-seven different variants were detected, 22 in *BRCA1* and 15 in *BRCA2,* which included three copy number variants (deletion of exon 22) in *BRCA1* (Table 1; Figure 2).

**TABLE 1:** Distribution of *BRCA1* and *BRCA2* pathogenic variants tested in the National Cancer Control Programme of the Ministry of Health for the years 2018–21 (n = 3,458).

No.	DNA [hg19] NM_007294.4 *BRCA1* NM_000059.3 *BRCA2*	Protein	rs number	No. of cases (% of all detected variants, n = 144)	% of total
**Distribution of *BRCA1* pathogenic variants tested for recurrent pathogenic variants for the Polish population (n = 3400)**					
1	c.5266dup	p.Gln1756Profs	rs80357906	63 (43.75)	1.82
2	c.181T>G	p.Cys61Gly	rs28897672	33 (22.92)	0.95
3	c.4035del	p.Glu1346fs	rs80357711	5 (3.47)	0.14
4	c.68_69del	p.Glu23fs	rs80357914	1 (0.69)	0.03
5	c.3695_3699del	p. Val1234fs	rs80357609	1 (0.69)	0.03
TOTAL				103 (71.53)	2.98
**Distribution of *BRCA1/2* pathogenic variants tested in selected patients using NGS panel (n = 260)**					
***BRCA1***					
1	c.4186C>T	p.Gln1396Ter	rs80357011	1 (0.69)	0.03
2	c.321del	p.Phe107fs	rs80357544	1 (0.69)	0.03
3	c.4689C>G	p.Tyr1563Ter	rs80357433	1 (0.69)	0.03
4	c.4986+4A>T	p.?	rs80358087	1 (0.69)	0.03
5	c.5030_5033del	p.Thr1677fs	-	1 (0.69)	0.03
6	exon 22 deletion	p.?	-	1 (0.69)	0.03
7	c.5346G>A	p.Trp1782Ter	rs80357284	1 (0.69)	0.03
8	c.5509T>G	p.Trp1837GLy	rs80356959	1 (0.69)	0.03
***BRCA2***					
9	c.1796_1800del	p.Ser599Ter	rs276174813	1 (0.69)	0.03
10	c.4483_4484del	p.Val1495fs	rs886038105	1 (0.69)	0.03
11	c.5851_5854del	p.Ser 1951fs	rs80359543	1 (0.69)	0.03
12	c.5946del	p.Ser 1982fs	rs80359550	1 (0.69)	0.03
13	c.6405_6409del	p.Asn2135fs	rs80359584	1 (0.69)	0.03
14	c.7007G>A	p.Arg2336His	rs28897743	1 (0.69)	0.03
15	c.7680dup	p.Gln2561fs	rs80359673	1 (0.69)	0.03
TOTAL				15 (10.42)	0.43
**Distribution of *BRCA1/2* pathogenic variants tested in selected patients using Sanger sequencing (n = 45**)					
***BRCA1***					
1	c.68_69del	p.Glu23fs	rs80357914	1 (0.69)	0.03
2	c.191G>A	p.Cys64Tyr	rs55851803	1 (0.69)	0.03
3	c.213-12A>G	-	rs80358163	2 (1.39)	0.06
4	c.302-1G>A	-	rs80358116	1 (0.69)	0.03
5	c.1510del	p.Arg504fs	rs80357908	1 (0.69)	0.03
6	c.1687C>T	p.Gln563Ter	rs80356898	2 (1.39)	0.06
7	c.4689C>G	p.Tyr1563Ter	rs80357433	1 (0.69)	0.03
8	c.4986+4A>T*	-	rs80358087	2 (1.39)	0.06
9	c.5030_5033del	p.Thr1677fs	rs80357580	1 (0.69)	0.03
***BRCA2***					
10	c.658_659del	p.Val220fs	rs80359604	1 (0.69)	0.03
11	c.3599_3600del	p.Cys1200Ter	rs80359391	2 (1.39)	0.06
12	c.3847_3848del	p.Val1283fs	rs80359405	1 (0.69)	0.03
13	c.6405_6409del	p.Asn2135fs	rs80359584	2 (1.39)	0.06
14	c.7007G>A	p.Arg2336His	rs28897743	1 (0.69)	0.03
15	c.7558C>T	p.Arg2520Ter	rs80358981	2 (1.39)	0.06
16	c.9253dupA	p.Thr3085fs	rs80359752	2 (1.39)	0.06
17	c.9371A>T	p.Asn3124Ile	rs28897759	1 (0.69)	0.06
TOTAL				24 (16.67)	0.69
**Distribution of *BRCA1/2* pathogenic variants tested in selected patients using MLPA panels (n = 13)**					
1	rsa 17q21(BRCA1ex22)x1	p.?	-	2 (1.39)	0.06
TOTAL				2 (1.39)	0.06

Screening for founder pathogenic variants in *BRCA1* revealed the presence of 5 out of 5 selected variants in 103 out of the 3,400 cancer patients and their selected relatives (3.03%). The c.5266dup variant was the most common and accounted for 61.16% of 5 tested *BRCA1* variants and 43.75% of all *BRCA1/2* variants detected, while c.181T>G accounted for 32.03% and 22.92%, respectively. The least frequently detected *BRCA1* changes were c.4035del (4.85% and 3.47%), c.68_69del (0.97% and 0.69%), and c.3695_3699del (0.97% and 0.69%) (Table 1). The frequency of the founder pathogenic *BRCA1* variants was 71.53% of all variants detected.

Among the 260 patients selected for NGS analysis, 15 pathogenic variants were detected (10.42% of all *BRCA1/2* variants detected). All changes were confirmed from the second blood sample using Sanger sequencing—not listed in a Sanger sequencing group (Table 1). One variant was revealed in the analysis of the CNVs.

Sanger sequencing and MLPA analyses have been offered to patients from families with the presence of the diagnosed pathogenic variant or patients with the somatic variant present in a cancer sample. These analyses have revealed 17 different pathogenic variants in 24 probands out of 45 patients tested using the Sanger sequencing and 1 variant in 2 related probands in the MLPA group of 13 patients (Table 1). In the Sanger sequencing group, 32 probands were first- or second-degree relatives of a pathogenic variant carrier (17 carriers discovered), 9 were diagnosed with a somatic variant in ovarian cancer tissue (4 germinal variants confirmed), and 4 had an earlier result from another laboratory (3 confirmed, 1 unconfirmed).

### Other findings

According to the National Cancer Control Programme of the Ministry of Health for the years 2018–21, also two *PALB2* variants [c.509_510del (p.Arg170Ilefs) and p.172_175del (p.Gln60Argfs)] and three *CHEK2* variants [c.1100delC (p.Thr367fs), g.27417113_27422508del, c.444+1G>A] were analysed for the 3,400 patients. We found *PALB2* variants in 19 (0.56%) patients (11 and 8, respectively) and *CHEK2* variants in 95 (2.79%) patients (15, 36, and 44, respectively). In this publication, we focused only on the high-penetrance susceptibility genes *BRCA1* and *BRCA2*.

## Discussion

According to The Organisation for Economic Cooperation and Development (OECD) Report 2021 in Poland, in 2020, breast cancer was the cause of 7,037 deaths and was the most frequent cancer among women (25%), while ovarian cancer accounted for 5% of all cancers (Commission, 2021). The 5-year cancer survival rate for breast cancer is still lower for our country than the European Union average of 77% *versus* 82%. One cause may be low participation in screening programmes, e.g., for breast (39.17% of the target population in 2019) (data from Lower Silesian Screening Coordination Centre), and the long time between the appearance of the first symptoms and diagnostics and treatment (Commission, 2021). Cascade testing of at-risk relatives of patients who carry a pathogenic variant may additionally help identify individuals who require specific anticancer prophylaxis (McAlarnen et al., 2021). In the light of these facts, there is a strong necessity to conduct screening for HBOC on a large scale in Poland, as well as in small towns where access to genetic counselling is limited. Family doctors should play a special role in this task.

Our results on the frequency of the founder pathogenic *BRCA1* variants which constitute 71.53% of all detected variants are consistent with a previous study on a Polish population (Table 2) (Kowalik et al., 2018; Cybulski et al., 2019). Overall, we revealed 37 different pathogenic variants in *BRCA1/2* genes. Our results demonstrate that a simple and inexpensive genetic test focusing on only five founder pathogenic variants can be successfully used as a rapid screening test in HBOC patients. Moreover, for the Lower Silesian population, the first-line test may be limited to only three variants in *BRCA1*: c.5266dup (p.Gln1756Profs), c.181T>G (p.Cys61Gly) and c.4035del (p.Glu1346fs).

**TABLE 2:** Comparison of test results from other regions of Poland obtained in other genetic laboratories in Poland.

		Number of tested samples	% Founder *BRCA1* variants
1	Kowalik et al. (2018)	2,931	64^a^
2	Cybulski et al. (2019)	1,018	84^b^
3	Our results	3,400	71.53

a Percentage was estimated with 14 VUS and 4 benign variants.

b Percentage was estimated with c.5251C>T (p.Arg1751Ter) and c.5346G>A (p.Trp1782Ter) (11 cases, 1.1% together).

Our study, like similar studies conducted in our country, showed that the prevalence of CNVs in *BRCA1/2* genes in the Polish population is lower than the average value observed worldwide. Only three patients from one family were carriers of CNVs (deletion of exon 22) in the *BRCA1* gene, which have been reported previously in European HBOC patients (Engert et al., 2008). Large genomic rearrangements (LGRs) involving *BRCA1/2* genes are rare in populations (and less frequently observed for *BRCA2*), but their detection is still important (Ewald et al., 2009). Their frequency varies from 0% to 28% in different populations (Engert et al., 2008; Ewald et al., 2009; El Ansari et al., 2020; Van Der Merwe et al., 2020). In our study group, 273 patients were tested for CNVs, and they accounted for 2.1% (3/144 patients with variants), although the CNV was familial. Combined diagnostics of SNVs and CNVs using NGS gene panels is the optimal solution in the care of HBOC patients.

Studies on large groups of patients and controls in the United States have revealed that pathogenic or likely pathogenic variants in critical HBOC genes are common both in patients meeting National Comprehensive Cancer Network (NCCN) criteria and in patients not meeting them—the difference in the prevalence of pathogenic and likely pathogenic variants, 9.39% and 7.9%, respectively, was not statistically significant (Beitsch et al., 2019). The advantage of universal genetic testing *versus* guideline-directed targeted testing was also supported by another independent study (Samadder et al., 2021). In the context of this research, it is reasonable to test for HBOC genes in all breast and ovarian cancer patients, irrespective of clinical data, as was applied in establishing the criteria for genetic tests analysing Polish recurrent HBOC-related mutations according to the National Cancer Control Programme of the Ministry of Health for the years 2018–21. Moreover, limiting testing to selected pathogenic variants should be carefully considered in relation to the possible founder effect (present in Poland) and the genetic structure of each population. In the era of NGS, limiting genetic tests seems to be illogical, and national health care in every country should strive to introduce such diagnostics (Dorling et al., 2021).

Genetic tests for *BRCA1/2* genes are also a cost-efficient screening tool for patients with somatic or germline pathogenic variants who can benefit from PARP inhibitor (PARPi) therapy (Forbes et al., 2019; McAlarnen et al., 2021; Szczerba et al., 2021; Yamamoto and Hirasawa 2021). Thus, *BRCA1/2* sequencing is more and more commonly ordered by oncologists as a test performed on tDNA not only for patients with ovarian cancer but also for specific patients with prostate cancer and pancreatic cancer in whom such therapy could be considered (McAlarnen et al., 2021; Szczerba et al., 2021). For such patients, a consultation with clinical geneticists still should be highly recommended and is crucial to determine whether the pathogenic variant is somatic or germline. Moreover, currently PARPi are also allowed for a specific group of patients with HER-2 negative breast cancer who are carriers of a germline *BRCA1/2* mutation, thus *BRCA1/2* sequencing on DNA isolated from blood or saliva could also be considered as a first-line genetic test in such cases (Yamamoto and Hirasawa 2021).

In the Polish population, the analysis for five founder pathogenic *BRCA1* variants as a first-line genetic test for HBOC seems to be logically and economically justified, which was also proved by our study. The question arises as to who from the group of subjects without a founder mutation should be referred for second-stage analysis, which, in the absence of other hot spots, should consist of *BRCA1/2* sequencing or even NGS multigene panel testing, which is particularly difficult in the absence of current official recommendations for HBOC genetic testing in our country. Another dilemma to consider is the scope of sequenced genes associated with HBOC in the context of ever decreasing costs and increasing availability of multigene NGS panels. The inclusion of high-penetrance HBOC-related genes such as *TP53*, *PALB2*, *PTEN*, *CDH1*, and *STK11* to the NGS panel seems to be justified, even considering the low prevalence of their germline pathogenic variants, as their identification significantly alters the level of cancer prophylaxis and can substantially impact therapeutic decisions in the patient.

It is also debatable whether this approach with optional second-stage analysis should be limited only to cases where there is no time pressure to obtain definitive results, because there is a risk of missing important clinical decisions that depend on the HBOC genetic test, such as the extent of surgical intervention in breast cancer patients. A strategy with an optional second step also runs the risk of situations such as missed carriers of non-founder pathogenic variants in *BRCA1/2* or other HBOC-related genes in individuals who did not meet the criteria for further genetic analysis or in whom no further testing was performed because of dropout or cancer-related death.

## Conclusion

Current observations show the increasing accessibility of multigene NGS panels and enlarging indications for the still growing number of therapies aimed at HR deficiency. The recommendations for genetic tests analysing somatic and germline pathogenic variants in patients with malignancies from the HBOC spectrum should therefore be constantly and consequently updated. In the face of this challenge, the increasing role of the molecular diagnostician and clinical geneticist in the multidisciplinary care of patients diagnosed with HBOC spectrum cancers is undeniable and necessary to provide complete and optimal therapeutic and preventive options consistent with current medical knowledge.

## Data Availability

The raw data supporting the conclusions of this article will be made available by the authors, without undue reservation.
