# Supplementary material for: Detection of BRCA1/2 pathogenic variants in patients with breast and/or ovarian cancer and their families. Analysis of 3,458 cases from Lower Silesia (Poland) according to the diagnostic algorithm of the National Cancer Control Programme
Source: Front Genet. 2022 Sep 12;13:941375. doi: 10.3389/fgene.2022.941375 (PMC9510890; doi:10.3389/fgene.2022.941375)
Supplement: Supplementary file 1 [file Table1.DOCX]

| BRCA1:c.3700_3704del (p.Val1234fs)  5ʹ FAM labelled  rs80357609, LRG_292t1 | (F) 5ʹ ACACTCGGTAGCAACGGT 3ʹ |
| --- | --- |
|  | (R) 5ʹ GCTTCCAACACTTGTTATTTGGT 3ʹ |
| BRCA1:c.68_69del (p.Glu23fs)  5ʹ FAM labelled  rs80357914, LRG_292t1 | (F) 5ʹ TCCCAAATTAATACACTCTTGTGC 3ʹ |
|  | (R) 5ʹ GCTCTTCGCGTTGAAGAAGTA 3ʹ |

Supplement Table. Sequences of the originally designed primers used for ASA-PCR.
